# The association between maternal diabetes and neonatal seizures: a nested case–Control study

**DOI:** 10.3389/fped.2023.1145443

**Published:** 2023-07-13

**Authors:** Yanjin Liang, Juman Liu, Xianbin Lin

**Affiliations:** Department of Pediatrics, Huizhou Central People's Hospital, Huizhou, China

**Keywords:** PGDM, GDM, neonatal seizure, PSM method, NVSS

## Abstract

**Aim:**

We aimed to evaluate the association of pregestational diabetes mellitus (PGDM) and gestational diabetes mellitus (GDM) with neonatal seizures during neonatal hospitalization.

**Methods:**

In this nested case–control study, all data were collected from the data files of the National Vital Statistics System (NVSS) 2016–2021. Considering the effect of confounders, we used the propensity-score matching (PSM; case:control = 1:4) method to select the study population. The outcome was considered the occurrence of neonatal seizures. Univariate and multivariate logistic regression analyses were adopted to assess the association of PGDM and GDM with neonatal seizures. We also conducted stratified analyses according to gestational age, birthweight, 5 min Apgar score, and maternal age to explore the potential disparities.

**Results:**

After using the PSM method, a total of 6,674 cases of neonatal seizures and 26,696 controls were included. After adjusting for covariates, PGDM was associated with an increased risk of neonatal seizures [odds ratio (OR) = 1.51, 95% confidence interval (CI): 1.15–1.98], whereas the association between GDM and neonatal seizures is not statistically significant. In addition, the correlation between PGDM and increased risk of neonatal seizures was observed in neonates with a gestational age of 37–42 weeks and ≥42 weeks, with a 5 min Apgar score of ≥7, and with a maternal age of ≤40 years.

**Conclusion:**

PGDM was found to be closely associated with an increased risk of neonatal seizures. The findings of our study indicated that neonatologists should consider monitoring the incidence of neonatal seizures in neonates born to mothers with PGDM.

## Introduction

Neonatal seizures are the most common neurological condition in newborns, and, depending on their etiology, can lead to long-term outcomes such as epilepsy, cerebral palsy, developmental disabilities, and psychomotor impairments (1, 2). The incidence of neonatal seizure is approximately 1.5–5.5 per 1,000 live births (2), which is considered a significant cause of neonatal mortality (3). Therefore, the identification of risk factors associated with neonatal seizures is crucial in reducing neurological morbidity and mortality among infants.

Previous studies have indicated that birth asphyxia may contribute to neonatal seizures and is associated with maternal complications both prior to and during delivery (4, 5). Recently, several studies have found a correlation between maternal diabetes, including pregestational diabetes mellitus (PGDM) and gestational diabetes mellitus (GDM), and the risk of neonatal seizure (6, 7). A retrospective cohort study assessed the relationship between neonatal complications and PGDM in infants born preterm (<36 weeks gestation), revealing that PGDM was associated with an elevated risk of seizures among neonates born <34 weeks gestation (6). After adjusting for these variables, Glass et al. found that both PGDM and GDM were risk factors for neonatal seizures, with PGDM having a greater impact (8). However, Hall et al. reported a relationship between PGDM and the increased risk of neonatal seizures, while no such association was found with GDM (9). To the best of our knowledge, existing studies on the relationship between maternal diabetes and neonatal seizures remain contentious. In addition, post-term delivery (≥42 weeks gestation) is also at high risk of developing neonatal seizures (8), but few studies have analyzed the relationship between maternal diabetes and neonatal seizures for post-term infants.

Herein, this study aims to evaluate the association of maternal diabetes and neonatal seizures during neonatal hospitalization in a large cohort of the National Vital Statistics System (NVSS).

## Methods

### Study population

We conducted a nested case–control study with data sources collected from the National Vital Statistics System (NVSS) 2016–2021 data files. The NVSS is the result of a partnership between the National Center for Health Statistics (NCHS) at the Centers for Disease Control and Prevention (CDC) and all US states, aiming to collect information on a wide range of maternal and infant demographic and health characteristics for all births (10, 11). This study is considered exempt from the review of the Huizhou Central People's Hospital Ethics Committee due to the use of deidentified data.

The inclusion criteria comprised (1) newborns diagnosed with seizures and (2) pregnant women with complete information about PGDM and GDM. The exclusion criteria were as follows: (1) the presence of multiple births and (2) participants with missing demographic information. A total of 20,297,340 participants met the criteria for this study (case: *n* = 20,290,666; control: *n* = 6,674). We used the propensity-score matching (PSM, case:control = 1:4) method to reduce the effects of gender, gestational age, and method of delivery. In total, 6,674 cases of neonatal seizures and 26,696 controls were ultimately included in this study.

### Pregestational diabetes mellitus and gestational diabetes mellitus

GDM is defined as glucose intolerance first detected during pregnancy (12), while PGDM is a condition where diabetes is diagnosed prior to conception (13).

### Outcome

Neonatal seizures were diagnosed by clinicians based on clinical criteria as follows: any involuntary repetitive, convulsive movement or behavior, and severe alteration of alertness such as obtundation, stupor, or coma. The primary outcome was the occurrence of neonatal seizures during neonatal hospitalization in this study.

### Potential covariates

Potential covariates were extracted as follows: maternal characteristics contain age (years), race, educational level, birthplace, previous cesarean delivery, previous preterm births, number of prenatal care visits, smoking before pregnancy, body mass index (BMI, kg/m^2^), weight gain (pounds), infertility treatment, pregestational hypertension, gestational hypertension, eclampsia, chorioamnionitis, infection, previous delivery, induction of labor, delivery method, use of anesthesia, steroids, and antibiotics. Infant characteristics contain gestational age, gender, birthweight (g), use of surfactant, and 5 min Apgar score.

### Statistical analysis

We used the mean ± standard deviation (mean ± SD) to describe the measurement data that conform to a normal distribution pattern, and for comparison between the case group and the control group, we used the *t*-test. The numerical data with non-normally distributed data were presented as a median and interquartile range [*M* (*Q*1, *Q*3)], and for comparison between the case group and the control group, the rank sum test was used. The categorical data were expressed as the number of cases and composition ratio [*n* (%)], and the Chi-square test was used for comparison between both groups.

Considering the effect of confounders, we used the PSM method in this study (14, 15). Participants with neonatal seizures were matched in a 1:4 ratio to those without. Subsequently, a descriptive analysis was conducted on the case and control groups both pre- and post-PSM. After performing PSM, univariate and multivariate logistic regression analyses were used to assess the association between maternal diabetes (contains PGDM and GDM) and neonatal seizure. Model 1 was not adjusted for covariates. Model 2 was adjusted for maternal age, birthplace, previous cesarean delivery, previous preterm births, number of prenatal care visits, smoking before pregnancy, BMI, weight gain, infertility treatment, pregestational hypertension, gestational hypertension, eclampsia, chorioamnionitis, infection, previous delivery, induction of labor, use of steroids and antibiotics, infant birthweight, use of surfactant, and 5 min Apgar score. It is worth mentioning that for investigating the relationship between GDM and neonatal seizures, Model 2 was further adjusted for PGDM based on the original adjusted covariates. In addition, we conducted stratified analyses based on gestational age, birthweight, 5 min Apgar score, and maternal age to explore the potential disparities in the association between maternal diabetes and neonatal seizures. The relationship between maternal diabetes and neonatal seizures was presented using an odds ratio (OR) with a 95% confidence interval (CI). A score of *P *< 0.05 was considered statistically significant. All statistical analyses were performed using SAS software (version 9.4; SAS Institute Inc., Cary, NC, USA) and R studio (version 4.2.1).

## Results

### Baseline characteristics

The baseline characteristics of the case and control groups, both pre- and post-PSM, are given in Table 1. After PSM, a total of 33,370 participants were selected for this study with 6,674 in the case group (neonatal seizure group) and 26,696 in the control group (non-neonatal seizure group). There were significant differences in some characteristics between the case and the control groups, such as maternal age, birthplace, previous cesarean delivery, previous preterm births, number of prenatal care visits, smoking before pregnancy, BMI, weight gain, infertility treatment, PGDM, pregestational hypertension, gestational hypertension, eclampsia, chorioamnionitis, infection, previous delivery, induction of labor, use of steroids and antibiotics, birthweight (g), use of surfactant, and 5 min Apgar score (*P *< 0.05). These may be potential covariates in this study.

**Table 1:** Baseline characteristics of the case and control groups before and after PSM.

Variables	Before PSM				After PSM			
	Total (*n* = 20,297,340)	Control group (*n* = 20,290,666)	Case group (*n* = 6,674)	*P*	Total (*n* = 33,370)	Control group (*n* = 26,696)	Case group (*n* = 6,674)	*P*
**Maternal characteristics**								
Age, years, mean ± SD	28.98 ± 5.79	28.98 ± 5.79	28.70 ± 6.13	<0.001	29.77 ± 6.02	30.04 ± 5.96	28.70 ± 6.13	<0.001
Race, *n* (%)				0.027				0.069
Other	5,287,190 (26.05)	5,285,531 (26.05)	1,659 (24.86)		8,585 (25.73)	6,926 (25.94)	1,659 (24.86)	
White	15,010,150 (73.95)	15,005,135 (73.95)	5,015 (75.14)		24,785 (74.27)	19,770 (74.06)	5,015 (75.14)	
Education level, *n* (%)				<0.001				0.784
High school or above	17,795,678 (87.67)	17,789,946 (87.68)	5,732 (85.89)		28,625 (85.78)	22,893 (85.75)	5,732 (85.89)	
Less than high school	2,501,662 (12.33)	2,500,720 (12.32)	942 (14.11)		4,745 (14.22)	3,803 (14.25)	942 (14.11)	
Birthplace, *n* (%)				<0.001				<0.001
In hospital	19,979,816 (98.44)	19,973,295 (98.44)	6,521 (97.71)		33,139 (99.31)	26,618 (99.71)	6,521 (97.71)	
Not in Hospital	317,524 (1.56)	317,371 (1.56)	153 (2.29)		231 (0.69)	78 (0.29)	153 (2.29)	
Previous cesarean delivery, *n* (%)				0.043				<0.001
No	17,165,385 (84.57)	17,159,681 (84.57)	5,704 (85.47)		25,445 (76.25)	19,741 (73.95)	5,704 (85.47)	
Yes	3,131,955 (15.43)	3,130,985 (15.43)	970 (14.53)		7,925 (23.75)	6,955 (26.05)	970 (14.53)	
Previous preterm births, *n* (%)				<0.001				<0.001
No	19,609,327 (96.61)	19,603,043 (96.61)	6,284 (94.16)		32,322 (96.86)	26,038 (97.54)	6,284 (94.16)	
Yes	688,013 (3.39)	687,623 (3.39)	390 (5.84)		1,048 (3.14)	658 (2.46)	390 (5.84)	
Number of prenatal care visits, *M* (*Q*1, *Q*3)	11.00 (9.00, 13.00)	11.00 (9.00, 13.00)	11.00 (8.00, 13.00)	<0.001	11.00 (9.00, 14.00)	12.00 (9.00, 14.00)	11.00 (8.00, 13.00)	<0.001
Smoking before pregnancy, *n* (%)				<0.001				<0.001
No	18,692,986 (92.10)	18,687,293 (92.10)	5,693 (85.30)		31,570 (94.61)	25,877 (96.93)	5,693 (85.30)	
Yes	1,604,354 (7.90)	1,603,373 (7.90)	981 (14.70)		1,800 (5.39)	819 (3.07)	981 (14.70)	
BMI, km/m^2^, mean ± SD	27.19 ± 6.72	27.19 ± 6.72	28.14 ± 7.29	<0.001	27.38 ± 6.81	27.19 ± 6.68	28.14 ± 7.29	<0.001
Weight gain, pounds, *M* (*Q*_1_, *Q*_3_)	29.00 (20.00, 38.00)	29.00 (20.00, 38.00)	29.00 (19.00, 39.00)	0.829	27.00 (19.00, 37.00)	27.00 (18.00, 36.00)	29.00 (19.00, 39.00)	<0.001
Infertility treatment, *n* (%)				<0.001				<0.001
No	19,971,774 (98.40)	19,965,304 (98.40)	6,470 (96.94)		32,763 (98.18)	26,293 (98.49)	6,470 (96.94)	
Yes	325,566 (1.60)	325,362 (1.60)	204 (3.06)		607 (1.82)	403 (1.51)	204 (3.06)	
PGDM, *n* (%)				<0.001				<0.001
No	20,102,348 (99.04)	20,095,850 (99.04)	6,498 (97.36)		32,879 (98.53)	26,381 (98.82)	6,498 (97.36)	
Yes	194,992 (0.96)	194,816 (0.96)	176 (2.64)		491 (1.47)	315 (1.18)	176 (2.64)	
GDM, *n* (%)				<0.001				0.690
No	18,890,424 (93.07)	18,884,305 (93.07)	6,119 (91.68)		30,635 (91.80)	24,516 (91.83)	6,119 (91.68)	
Yes	1,406,916 (6.93)	1,406,361 (6.93)	555 (8.32)		2,735 (8.20)	2,180 (8.17)	555 (8.32)	
Pregestational hypertension, *n* (%)				<0.001				<0.001
No	19,864,631 (97.87)	19,858,238 (97.87)	6,393 (95.79)		32,666 (97.89)	26,273 (98.42)	6,393 (95.79)	
Yes	432,709 (2.13)	432,428 (2.13)	281 (4.21)		704 (2.11)	423 (1.58)	281 (4.21)	
Gestational hypertension, *n* (%)				<0.001				<0.001
No	18,823,140 (92.74)	18,817,260 (92.74)	5,880 (88.10)		30,598 (91.69)	24,718 (92.59)	5,880 (88.10)	
Yes	1,474,200 (7.26)	1,473,406 (7.26)	794 (11.90)		2,772 (8.31)	1,978 (7.41)	794 (11.90)	
Eclampsia, *n* (%)				<0.001				<0.001
No	20,245,765 (99.75)	20,239,149 (99.75)	6,616 (99.13)		33,251 (99.64)	26,635 (99.77)	6,616 (99.13)	
Yes	51,575 (0.25)	51,517 (0.25)	58 (0.87)		119 (0.36)	61 (0.23)	58 (0.87)	
Chorioamnionitis, *n* (%)				<0.001				<0.001
No	19,972,786 (98.40)	19,966,522 (98.40)	6,264 (93.86)		32,358 (96.97)	26,094 (97.74)	6,264 (93.86)	
Yes	324,554 (1.60)	324,144 (1.60)	410 (6.14)		1,012 (3.03)	602 (2.26)	410 (6.14)	
Infection, *n* (%)				<0.001				<0.001
No	19,860,779 (97.85)	19,854,434 (97.85)	6,345 (95.07)		32,694 (97.97)	26,349 (98.70)	6,345 (95.07)	
Yes	436,561 (2.15)	436,232 (2.15)	329 (4.93)		676 (2.03)	347 (1.30)	329 (4.93)	
Previous delivery, *n* (%)				<0.001				<0.001
No	7,907,123 (38.96)	7,903,543 (38.95)	3,580 (53.64)		14,380 (43.09)	10,800 (40.46)	3,580 (53.64)	
Yes	12,390,217 (61.04)	12,387,123 (61.05)	3,094 (46.36)		18,990 (56.91)	15,896 (59.54)	3,094 (46.36)	
Induction of labor, *n* (%)				<0.001				<0.001
No	14,350,695 (70.70)	14,346,165 (70.70)	4,530 (67.88)		26,946 (80.75)	22,416 (83.97)	4,530 (67.88)	
Yes	5,946,645 (29.30)	5,944,501 (29.30)	2,144 (32.12)		6,424 (19.25)	4,280 (16.03)	2,144 (32.12)	
Use of anesthesia, *n* (%)				<0.001				0.072
No	4,865,841 (23.97)	4,864,044 (23.97)	1,797 (26.93)		8,696 (26.06)	6,899 (25.84)	1,797 (26.93)	
Yes	15,431,499 (76.03)	15,426,622 (76.03)	4,877 (73.07)		24,674 (73.94)	19,797 (74.16)	4,877 (73.07)	
Use of steroids, *n* (%)				<0.001				<0.001
No	19,738,917 (97.25)	19,732,711 (97.25)	6,206 (92.99)		31,508 (94.42)	25,302 (94.78)	6,206 (92.99)	
Yes	558,423 (2.75)	557,955 (2.75)	468 (7.01)		1,862 (5.58)	1,394 (5.22)	468 (7.01)	
Use of antibiotics, *n* (%)				<0.001				<0.001
No	15,235,618 (75.06)	15,231,351 (75.07)	4,267 (63.93)		22,366 (67.02)	18,099 (67.80)	4,267 (63.93)	
Yes	5,061,722 (24.94)	5,059,315 (24.93)	2,407 (36.07)		11,004 (32.98)	8,597 (32.20)	2,407 (36.07)	
Delivery method, *n* (%)				<0.001				1.000
Spontaneous	13,465,828 (66.34)	13,463,081 (66.35)	2,747 (41.16)		13,735 (41.16)	10,988 (41.16)	2,747 (41.16)	
Forceps	106,594 (0.53)	106,488 (0.52)	106 (1.59)		530 (1.59)	424 (1.59)	106 (1.59)	
Vacuum	530,461 (2.61)	530,096 (2.61)	365 (5.47)		1,825 (5.47)	1,460 (5.47)	365 (5.47)	
Cesarean	6,194,457 (30.52)	6,191,001 (30.51)	3,456 (51.78)		17,280 (51.78)	13,824 (51.78)	3,456 (51.78)	
**Infant characteristics**								
Gestational age, *n* (%)				<0.001				1.000
<37	2,007,729 (9.89)	2,006,299 (9.89)	1,430 (21.43)		7,150 (21.43)	5,720 (21.43)	1,430 (21.43)	
37–42	17,237,652 (84.93)	17,232,790 (84.93)	4,862 (72.85)		24,310 (72.85)	19,448 (72.85)	4,862 (72.85)	
≥42	1,051,959 (5.18)	1,051,577 (5.18)	382 (5.72)		1,910 (5.72)	1,528 (5.72)	382 (5.72)	
Gender, *n* (%)				<0.001				1.000
Female	9,911,702 (48.83)	9,908,773 (48.83)	2,929 (43.89)		14,645 (43.89)	11,716 (43.89)	2,929 (43.89)	
Male	10,385,638 (51.17)	10,381,893 (51.17)	3,745 (56.11)		18,725 (56.11)	14,980 (56.11)	3,745 (56.11)	
Combined gestation, week, Mean ± SD	38.72 ± 2.28	38.72 ± 2.28	37.91 ± 3.48	<0.001	38.12 ± 3.02	38.18 ± 2.89	37.91 ± 3.48	<0.001
Birthweight, g, mean ± SD	3,297.56 ± 553.26	3,297.62 ± 553.15	3,103.68 ± 790.85	<0.001	3,187.30 ± 692.95	3,208.20 ± 664.60	3,103.68 ± 790.85	<0.001
Use of surfactant, *n* (%)				<0.001				<0.001
No	20,221,797 (99.63)	20,215,783 (99.63)	6,014 (90.11)		32,529 (97.48)	26,515 (99.32)	6,014 (90.11)	
Yes	75,543 (0.37)	74,883 (0.37)	660 (9.89)		841 (2.52)	181 (0.68)	660 (9.89)	
5 min Apgar score, score, *M* (*Q*_1_, *Q*_3_)	9.00 (9.00, 9.00)	9.00 (9.00, 9.00)	7.00 (3.00, 9.00)	<0.001	9.00 (9.00, 9.00)	9.00 (9.00, 9.00)	7.00 (3.00, 9.00)	<0.001

PSM, propensity-score matching; BMI, body mass index; PGDM, pregestational diabetes mellitus; GDM, gestational diabetes mellitus; SD, standard deviation; *M* (*Q*1, *Q*3), median and interquartile range.

### The association of maternal diabetes and neonatal seizures

Table 2 shows an association between maternal diabetes and neonatal seizures. In unadjusted analysis, PGDM was found to be a risk factor for neonatal seizures (OR = 2.27, 95% CI: 1.88–2.73, *P *< 0.001). After adjusting for all covariates, PGDM was still associated with an increased risk of neonatal seizures (OR = 1.51, 95% CI: 1.15–1.98, *P *= 0.003). In addition, we observed that, after adjusting for covariates, the association between GDM and neonatal seizure was not statistically significant (*P *= 0.405).

**Table 2:** The association of maternal diabetes and neonatal seizures.

Variables	Model 1		Model 2	
	OR (95% CI)	*P*	OR (95% CI)	*P*
PGDM				
No	Ref		Ref	
Yes	2.27 (1.88–2.73)	<0.001	1.51 (1.15–1.98)*	0.003
GDM				
No	Ref		Ref	
Yes	1.04 (0.94–1.14)	0.468	1.06 (0.92–1.21)#	0.405

PGDM, pregestational diabetes mellitus; GDM, gestational diabetes mellitus; OR, odds ratio; CI, confidence interval.

Model 1: not adjusted for covariates;

Model 2:

* Adjusted maternal age, birthplace, previous cesarean delivery, previous preterm births, number of prenatal care visits, smoking before pregnancy, body mass index (BMI), weight gain, infertility treatment, pregestational hypertension, gestational hypertension, eclampsia, chorioamnionitis, infection, previous delivery, induction of labor, use of steroids and antibiotics, infant birthweight, use of surfactant, and 5 min Apgar score.

# Adjusted PGDM, maternal age, birthplace, previous cesarean delivery, previous preterm births, number of prenatal care visits, smoking before pregnancy, BMI, weight gain, infertility treatment, pregestational hypertension, gestational hypertension, eclampsia, chorioamnionitis, infection, previous delivery, induction of labor, use of steroids and antibiotics, infant birthweight, use of surfactant, and 5 min Apgar score.

### Subgroup analysis based on gestational age, birthweight, 5 min Apgar score, and maternal age

**Figure 1:**
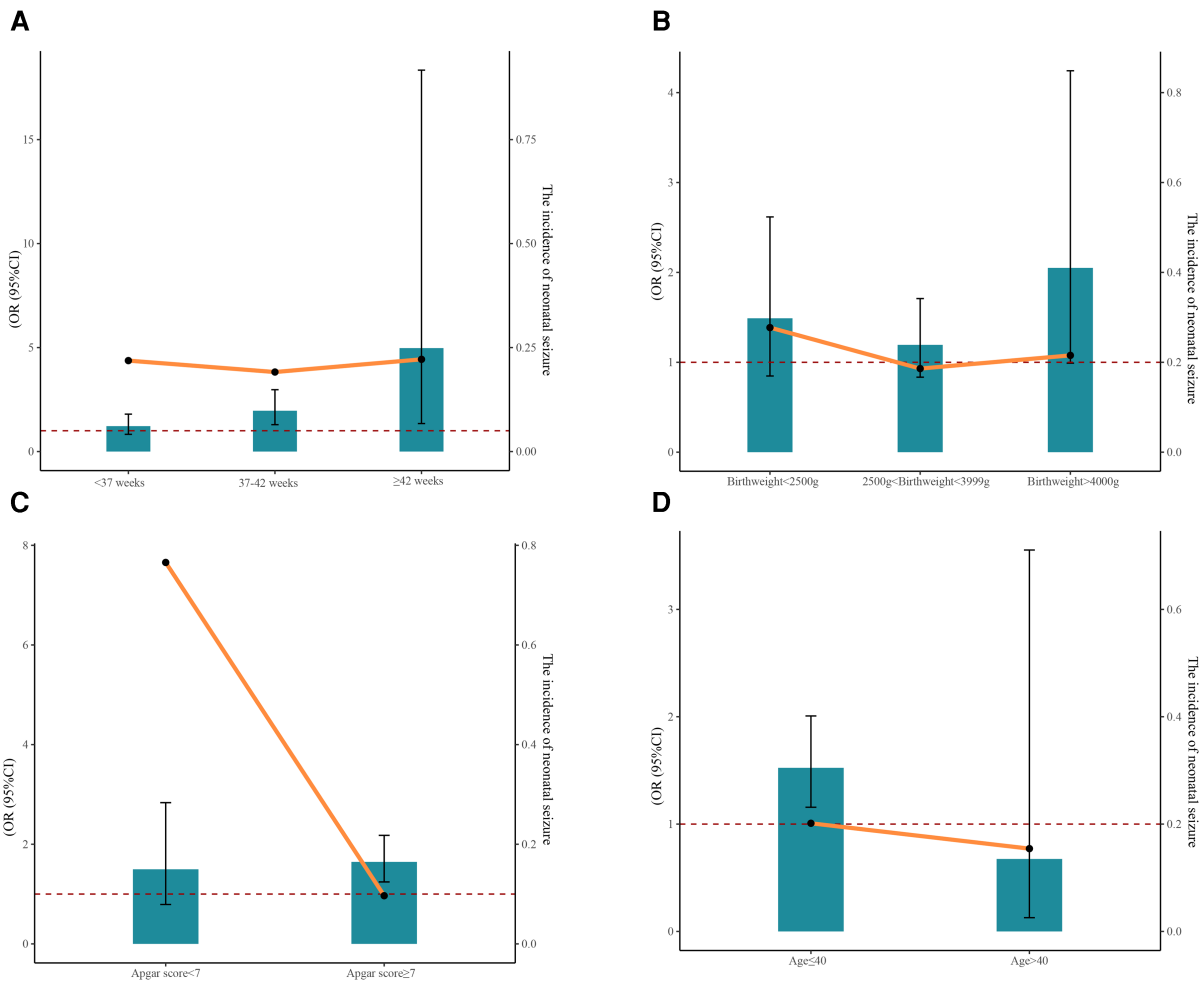
The incidence of neonatal seizure and the 95% CI of OR based on gestational age (**A**), birthweight (**B**), Apgar score (**C**), and maternal age (**D**).

The results of stratified analyses based on gestational age, birthweight, 5 min Apgar score, and maternal age are displayed in Table 3. A correlation between PGDM and increased risk of neonatal seizures was observed for neonates with a gestational age of 37–42 weeks (Model 2: OR = 1.82, 95% CI: 1.24–2.66, *P *= 0.002) and ≥ 42 weeks (Model 2: OR = 4.97, 95% CI: 1.35–18.34, *P *= 0.016). There was no statistically significant association between PGDM or GDM and neonatal seizures in neonates of varying birthweights. Among neonates possessing different Apgar scores, PGDM was a risk factor for neonatal seizure only when the Apgar score was ≥7 (Model 2: OR = 1.65, 95% CI: 1.24–2.18, *P *< 0.001). Furthermore, we found that PGDM was associated with an increased risk of neonatal seizures among maternal age ≤40 (Model 2: OR = 1.52, 95% CI: 1.16–2.01, *P *= 0.003). Figure 1 also depicts the incidence of neonatal seizures and the OR with a 95% CI based on gestational age, birthweight, Apgar score, and maternal age.

**Table 3:** Stratified analyses based on gestational age, birthweight, Apgar score, and maternal age.

Stratified analyses		Model I		Model II	
		OR (95% CI)	*P*	OR (95% CI)	*P*
**Gestational age, years**					
<37	PGDM (Yes)	1.21 (0.82–1.78)	0.339	1.22 (0.83–1.80)	0.312
	GDM (Yes)	0.89 (0.69–1.16)	0.395	0.93 (0.71–1.20)	0.564
37–42	PGDM (Yes)	1.77 (1.21–2.60)	0.003	1.82 (1.24–2.66)	0.002
	GDM (Yes)	1.00 (0.84–1.18)	0.974	1.06 (0.90–1.26)	0.490
≥42	PGDM (Yes)	4.99 (1.36–18.36)	0.016	4.97 (1.35–18.34)	0.016
	GDM (Yes)	0.86 (0.43–1.72)	0.671	0.86 (0.43–1.72)	0.666
**Birthweight, g**					
<2,500	PGDM (Yes)	1.19 (0.69–2.07)	0.535	1.49 (0.85–2.62)	0.166
	GDM (Yes)	1.10 (0.80–1.50)	0.556	1.19 (0.86–1.65)	0.290
2,500–4,000	PGDM (Yes)	1.09 (0.76–1.55)	0.650	1.19 (0.83–1.71)	0.333
	GDM (Yes)	0.89 (0.75–1.04)	0.152	0.96 (0.82–1.14)	0.656
>4,000	PGDM (Yes)	1.88 (0.92–3.84)	0.086	2.05 (0.99–4.24)	0.053
	GDM (Yes)	0.87 (0.55–1.36)	0.539	0.90 (0.57–1.41)	0.639
**5 min Apgar score, score**					
<7	PGDM (Yes)	1.00 (0.52–1.91)	0.999	1.50 (0.79–2.83)	0.216
	GDM (Yes)	0.77 (0.54–1.10)	0.151	0.90 (0.63–1.30)	0.580
≥7	PGDM (Yes)	1.50 (1.13–1.98)	0.005	1.65 (1.24–2.18)	<0.001
	GDM (Yes)	1.00 (0.87–1.15)	0.986	1.08 (0.93–1.24)	0.309
**Maternal age, years**					
≤40	PGDM (Yes)	1.28 (0.97–1.69)	0.076	1.52 (1.16–2.01)	0.003
	GDM (Yes)	0.97 (0.84–1.11)	0.611	1.02 (0.89–1.17)	0.745
>40	PGDM (Yes)	0.47 (0.08–2.65)	0.393	0.67 (0.13–3.55)	0.643
	GDM (Yes)	0.94 (0.49–1.77)	0.838	0.99 (0.52–1.89)	0.973

PGDM, pregestational diabetes mellitus; GDM, gestational diabetes mellitus; OR, odds ratio; CI, confidence interval.

Model I adjusted for birthplace, previous cesarean delivery, previous preterm births, number of prenatal care visits, smoking before pregnancy, body mass index (BMI), weight gain, infertility treatment, pregestational hypertension, gestational hypertension, eclampsia, chorioamnionitis, infection, previous delivery, induction of labor, use of steroids and antibiotics, infant birthweight, use of surfactant, and 5 min Apgar score.

Model II adjusted for gestational age, maternal age, birthplace, previous cesarean delivery, previous preterm births, number of prenatal care visits, smoking before pregnancy, BMI, weight gain, infertility treatment, pregestational hypertension, gestational hypertension, eclampsia, chorioamnionitis, infection, previous delivery, induction of labor, use of steroids and antibiotics, infant birthweight, use of surfactant, and 5 min Apgar score.

For stratified analysis of gestational age, Model II was not adjusted for gestational age.

For stratified analysis of birthweight, Model I and Model II were not adjusted for infant birthweight.

For stratified analysis of the 5 min Apgar score, Model I and Model II were not adjusted for the 5 min Apgar score.

For stratified analysis of maternal age, Model II was not adjusted for maternal age.

In the stratified analysis of the relationship between GDM and neonatal seizures, Model I and Model II were adjusted for PGDM.

## Discussion

This nested case–control study analyzed the representative NVSS database to investigate the relationship between maternal diabetes and neonatal seizures. The findings indicated that PGDM was associated with an elevated risk of neonatal seizures.

The risk of neonatal seizures was the highest during the first month after birth (16), suggesting that multiple obstetric risk factors may influence their occurrence (17, 18). Previous studies have assessed the impact of maternal diabetes on neonatal seizures (6, 7). However, the conclusions regarding the relationship between maternal diabetes and neonatal seizures are controversial. In recent years, the PSM statistical method has gained widespread popularity because of its ability to balance differences between groups and reduce the influence of confounding variables (19, 20). In this study, we included 6,674 cases of neonatal seizures and 26,696 controls by using the PSM method. After performing PSM, a significantly higher proportion of mothers diagnosed with PGDM were observed in the neonatal seizure group compared with those in the non-neonatal seizure group (*P *< 0.001). After adjusting for all covariates, PGDM was considered to be related to an increased risk of neonatal seizures (OR = 1.51, 95% CI: 1.15–1.98), which was in keeping with previous studies (6, 8). This study also found that PGDM was a risk factor for neonatal seizures for neonates with a gestational age of ≥37 weeks. This finding is inconsistent with the result reported by Tse et al. (6). This may be attributed to the presence of different research subjects. In addition, preterm birth was associated with an elevated risk of neonatal seizures (21). The impact of preterm birth on the risk of neonatal seizures may be greater than that of PGDM in this study, thus rendering the association between PGDM and neonatal seizures insignificant within the preterm population. Similarly, this study found a significant association between PDGM and neonatal seizures for neonates with an Apgar score of ≥7 and a maternal age of ≤40 years. A low 5 min Apgar score and advanced maternal age were considered perinatal risk factors for infantile seizures (22, 23). We speculated that neonatal seizure risk may be more influenced by a low Apgar score (<7) and gestational age (>40 years), potentially obscuring the impact of PGDM. Additional samples will be collected from our hospitals in the future to further validate this finding and investigate the potential underlying mechanisms. It is noteworthy that birthplace was a confounding factor in this study. A retrospective cohort study has demonstrated that neonates who are delivered outside of a hospital setting have an increased risk of experiencing seizures when compared with those who are delivered in-hospital, as planned (24).

To our knowledge, the main causes of neonatal seizures are hypoglycemia, ischemia, structural lesions or abnormalities, and infections (9, 25). Notably, hypoglycemia frequently occurs in newborns born to mothers with PGDM (26). In this study, we found no statistically significant association between GDM and neonatal seizures. It has been widely reported that PGDM is associated with a higher risk of adverse pregnancy outcomes compared with GDM (27, 28). Given the critical period of organogenesis during early pregnancy, prolonged exposure to prepregnancy hyperglycemia and intrauterine hyperglycemia may elevate the likelihood of neonatal seizures (29, 30). The underlying mechanism regarding the association of PGDM, GDM, and neonatal seizure risk remains unclear. More research is needed in the future to clarify the mechanisms by which PGDM affects neonatal seizure risk.

Some limitations of this study need to be taken into account. First, this study was retrospective in nature, which may have led to selection bias. However, we employed the PSM statistical method to mitigate confounding effects and enhance the reliability of our findings. Second, our investigation solely focuses on the correlation between maternal diabetes and neonatal seizures during hospitalization, with no knowledge of seizure occurrence after discharge; moreover, the observation period in this study was less than 1 month. These factors may result in an underestimation of neonatal seizure incidence. Third, the study recruited participants from the NVSS database, which did not contain records of prenatal brain ultrasound magnetic resonance imaging, electroencephalography or amplitude-integrated electroencephalography, glycosylated hemoglobin during pregnancy information, and the Score for Neonatal Acute Physiology II with Perinatal Extension at admission. Last, it should be noted that the NVSS database lacks information on the specific type of PGDM, thus necessitating further investigation into the association between type 1 and type 2 diabetes and neonatal seizures.

## Conclusion

In short, this study revealed an association between PGDM and an elevated risk of neonatal seizures. Therefore, it is recommended that neonatologists closely monitor the incidence of seizures in newborns born to mothers with PGDM.

## Data Availability

Publicly available datasets were analyzed in this study. These data can be found here: Centers for Disease Control and Prevention (CDC) National Vital Statistics System (NVSS) database, https://www.cdc.gov/nchs/nvss/index.htm.
